# Vitamin D Status Is Associated with Disease Activity among Rheumatology Outpatients

**DOI:** 10.3390/nu5072268

**Published:** 2013-06-26

**Authors:** Zohreh Sabbagh, Janet Markland, Hassanali Vatanparast

**Affiliations:** 1 Department of Internal Medicine, College of Medicine, University of Saskatchewan, Saskatoon, SK S7J 5B6, Canada; E-Mails: zos852@mail.usask.ca (Z.S.); jmakland@sasktel.net (J.M.); 2 Division of Nutrition and Dietetics, College of Pharmacy and Nutrition, School of Public Health, University of Saskatchewan; Saskatoon SK S7N 5C9, Canada

**Keywords:** rheumatic disease, rheumatoid arthritis, vitamin D, inflammation, disease activity

## Abstract

The co-existence of high prevalence of vitamin D inadequacy among Canadians and high prevalence of systematic autoimmune rheumatic diseases (SARDs) raise the question on relationship between the two situations. Objective: To determine vitamin D status in known cases of common SARDs and compare to those with non-autoimmune diseases; further, to evaluate the impact of vitamin D on disease activity in rheumatoid arthritis (RA) cases. Methods: In a retrospective case-control study design, we evaluated 116 patients in a community clinic classified in two groups, Control group: patients with non-rheumatic disease (*n =* 56), and Case group: those with rheumatic diseases (*n =* 60). We compared plasma vitamin D status (25(OH)D), indicators of disease activity and other potential confounders. Further, we determined factors associated with disease activity in RA cases. Results: The plasma 25(OH)D was significantly lower in Case group (64.8 ± 29.8) compared to Control group (86.8 ± 37.7). High number of SARDs outpatients 56%) had considerably low plasma 25(OH)D concentration. RA cases with low plasma 25(OH)D had over five times higher risk of disease activity (OR *=* 5.15 95% CI 1.16, 22.9; *p =* 0.031). Conclusion: Inadequate vitamin D status in SARDs cases, along with considerably strong association with disease activity in RA cases, indicate the need for proper evaluation of vitamin D status in this clinical population. Moreover, appropriate training should be given to the patients to ensure the intake of the recommended amount of vitamin D per day through diet or supplement.

## 1. Introduction

Systematic autoimmune rheumatic disorders (SARDs) are a cluster of chronic autoimmune disorders associated with significant mortality and morbidity in developed countries, including Canada [1]. Rheumatoid arthritis (RA) as the most common disease in this group, affects around 0.9% of Canadian population; which will increase to 1.3% over the next 30 years [2]. The chronic progressive nature of the disease disables over 50% of cases within 10 years if not treated properly [2]. Early diagnosis and proper intervention strategies are key factors in managing the SARDs and decreasing the burden of disease. Since the prevalence of SARDs is affected by genetic and environmental factors [3], nutrition intervention might have impact in disease prevention and risk reduction.

The co-existence of a high prevalence of vitamin D inadequacy among Canadians, particularly in long winters [4], and the high prevalence of SARDs may raise the question on the relationship between these two. Vitamin D is traditionally known for its role in bone mineral homeostasis [5]. However, recent research reveals the existence of vitamin D receptors (VDR) in a variety of cells including anti-presenting cells (APC) [3,5,6]. This indicates the influence of vitamin D in various physiologic processes; one of them is the immune system. Active metabolite of vitamin D (1,25(OH)2D) inhibits the synthesis of IL-1, IL-6, IL-12 and TNF-α by macrophages [7]. It also decreases MHC-II expression on cell surface and molecules such as CD86, CD80 and CD 40 [3,7]. Finally, it increases apoptosis induced by DC and T lymphocytes. Animal studies report the role of vitamin D in preventing autoimmune encephalomyelitis, systemic lupus erythematosus (SLE), collagen-induced arthritis, and inflammatory bowel disease [7]. Along with these findings, evidence suggests an increase in the emergence of self-reactive T cells where the development of immune system co-exists with low vitamin D status [7]. All this evidence suggests vitamin D deficiency might trigger an autoimmune response and appropriate vitamin D status presents immunosuppressive effect [7].

A systematic review of studies on vitamin D and SARDs reported, comparing to healthy control groups, many case-control studies found lower vitamin D status in SARDs cases including SLE, RA, ankylosing spondylitis, scleroderma, Type 1 diabetes, Multiple Sclerosis and Crohn’s disease [8]. Further, current evidence from mainly small scale heterogeneous studies indicate possible role of vitamin D in improving disease activity in SARD cases such as RA, Multiple Sclerosis, type 1 diabetes, SLE and Crohn’s disease [8]. The association between vitamin D status and diseases activity in RA was evaluated in a recent meta-analyses of current studies including data from three cohort, six cross-sectional and two case-control studies (*n =* 215,757) [9]. Song *et al.* report inverse association between RA disease activity and serum vitamin D levels [9]. Recent advances in the role of vitamin D in various diseases and very limited dietary sources has promoted the intake of vitamin D, particularly in Canada’s high latitude areas with long winters such as Saskatoon [4,10], where vitamin D synthesis through sun is limited for at least seven months of the year. However, whether adult patients diagnosed with common autoimmune diseases in Canada have adequate levels of serum vitamin D is not known. The purpose of this study is to determine serum vitamin D status of known cases of common autoimmune diseases and compare to those with non-autoimmune diseases; also to evaluate the impact of vitamin D on disease activity.

## 2. Materials and Methods

In a retrospective case-control study design, we evaluated the charts of over 4000 patients and selected 116 patients with measured plasma 25-hydoxy vitamin D (25(OH)D). Using information on final diagnosis we categorized patients in case and control groups; 60 patients with autoimmune rheumatologic disease, as well as 56 patients with non-autoimmune conditions who were visited in one of the private rheumatology clinics in Saskatoon from January 2010 to the end of December 2010. Information on age, sex, BMI, plasma 25(OH)D, vitamin D and calcium supplement use, serum calcium, serum phosphate, erythrocyte sedimentation rate (ESR), C-reactive protein (CRP), glomerular filtration rate (GFR) and season were collected and recorded. We also collected data on disease activity score (DAS 28-ESR) only in cases with rheumatoid arthritis. DAS28-ESR is a quantitative measure of disease activity in rheumatoid arthritis, calculated by using a formula that considers the number of tender joints and swollen joints within 28 joints, as well as ESR [11]. The disease activity is considered high with the score of >5.1, low with <3.2 score, and the score of <2.6 for diseases in remission [11].

Independent *t*-test was used to evaluate the differences between patients in case and control group. Pearson correlation was used to determine the relationship between disease activity and serum vitamin D status in RA cases. We used the threshold of 50 nmol/L to define vitamin D deficiency based on recent literature [6,12]. To evaluate distribution of cases and controls across vitamin D status groups (deficient and optimal), Chi-square test was applied. Logistic regression was used to identify the association between vitamin D status and disease (autoimmune *vs.* non-auto immune). Data manipulation, cleaning, and creation of new variables and statistical analyses were done using SPSS IBM (Version 19, Armonk, NY, USA). In all analyses, alpha was set at the level of 0.05. Ethics approval was obtained from the Royal University Hospital Ethics Board, at the University of Saskatchewan.

## 3. Results

The characteristics of participants in case and control groups are presented in Table 1. In the control group, most patients were suffering from osteoarthritis, osteoporosis, fibromyalgia and gout, in descending order. In case group, rheumatoid arthritis (*n =* 39) was the most common autoimmune disease. Other autoimmune diseases included SLE, Sjogren, mixed connective tissue disease (MCTD), Wegner, psoriasis arthritis, and scleroderma.

**Table 1:** Characteristics of participants in Case and Control groups.

	Case group (*n =* 60)		Control group (*n =* 56)	
	Mean	SD	Mean	SD
Age *	54.5	13.0	65.0	11.7
BMI	29.0	8.1	29.7	5.4
Total 25 vitamin D value (nmol/L) *	64.8	29.8	86.8	37.7
Vitamin D supplement use (IU)	1043.6	649.4	1008.0	664.7
Serum Calcium *	2.3	0.1	2.4	0.1
Calcium supplement use (mg/day) *	459.3	439	715.1	591
Pho4	1.0	0.2	1.1	0.2
ESR	23.68	20.5	19.18	14.6
CRP *	12.9	19.3	6.07	9.6
Creatinine	72.1	44.4	78.8	33.9
GFR	69.9	28.7	71.6	20.5

* Significant difference (*p <* 0.05), independent student *t*-test.

**Figure 1:**
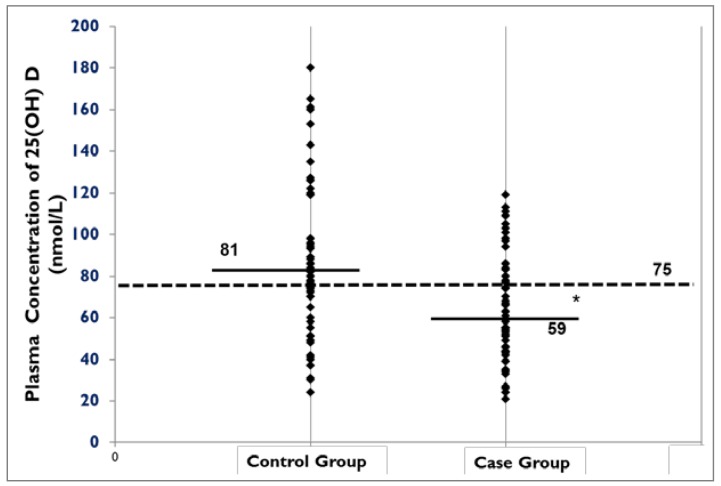
Plasma concentration of 25(OH) D across case and control groups.

Patients in case group were significantly younger. Only 13 participants were males (3 in control group and 10 in case group). Although most patients in both case (91.7%) and control (83.9%) groups reported taking approximately 1000 IU vitamin D supplement intake (median of 1000 IU in both case and control group), the mean plasma 25(OH)D was significantly lower in patients with autoimmune rheumatic diseases compared to control patients (*p <* 0.05) (Figure 1). Also, serum calcium was lower in case group (*p =* 0.01). In case group, 91.7% of participants reported vitamin D supplement use, among them 47.5% had vitamin D supplement prescription. The significantly higher CRP in patients with autoimmune rheumatic disease may indicate the higher disease activity in those patients (*p =* 0.035). A negative correlation was observed between plasma 25(OH)D and CRP with a borderline significance, probably due to a small sample size (*r =* −0.2, *p =* 0.087).

In the control group 11 patients 19.6%) with no autoimmune diseases were vitamin D deficient (plasma 25(OH)D < 50 nmol/L). Whereas in the case group, 20 patients (34.5%) were vitamin D deficient. The distribution of patients across plasma 25(OH)D groups (deficient, optimal) presented a borderline significant difference (*p =* 0.05) between case and control groups with higher distribution of patients with autoimmune diseases in deficient vitamin D group.

In all subjects, serum vitamin D was significantly higher in those who were measured in summer compared to the winter months (85.1 ± 33.4 *vs.* 70.8 ± 35.9, *p <* 0.05). While no significant difference in plasma 25(OH)D was observed by season in control group, plasma 25(OH)D was significantly higher in summer compared to the winter months in case group (82.2 ± 30.3 *vs.* 61.9 ± 32.7, *p =* 0.019). Evaluating the seasonal difference between case and control groups showed no significant difference in plasma 25(OH)D between case and control groups in summer. However, in winter, plasma 25(OH)D was significantly lower in case group compared to the control group (52.3 ± 22.4 *vs.* 86.0 ± 36.3, *p =* 0.02, in case and control groups respectively).

A considerable proportion of RA cases (59.6%) had a disease activity score of above 2.6 (cut off for disease remission). Further, the significant negative correlation between plasma 25(OH)D and disease activity (*r =* −0.43, *p =* 0.01) may indicate lower disease activity with increase in plasma 25(OH)D. In logistic regression analyses to evaluate the association between vitamin D status and disease activity in RA cases, adjusted for age, sex and season, the odds of having active disease was 5.15 times higher in patients with low plasma 25(OH)D compared to those with adequate vitamin D (O*R =* 5.15 95% CI 1.16, 22.9; *p =* 0.031).

## 4. Discussion

Our study is the first Canadian study to evaluate the vitamin D status of rheumatology outpatients in two distinct groups: those who are suffering from SARD in comparison to non-SARD patients. We found significantly low plasma 25(OH)D in SARD group compared to non-SARD group despite similar self-reported vitamin D supplement use, as well as seasonal difference in plasma 25(OH)D concentration in SARD cases. Further, among RA cases, considerably higher risk of disease activity in individuals with low 25(OH)D concentration is in agreement with other studies suggesting disturbance in vitamin D metabolism in RA and association between vitamin D and disease activity in rheumatic patients [8,9,13,14,15,16,17].

The low plasma 25(OH)D concentration (<75 nmol/L) in a considerable number of patients with SARD (56%) and its relationship with indicators of disease activity justifies the need for thorough evaluation of vitamin D status in this at-risk population in clinical settings. In our study, only 2.9% of patients with SARD and non-SARD diseases, who were visited in a community clinic, were evaluated for vitamin D status. Osteoporotic patients, as one of the common non-SARD cases visited in rheumatology clinics, receive clear instructions on vitamin D intake as adjunct therapy as indicated by Osteoporosis Canada [18]. This might explain why the influence of season on serum vitamin D likely has been minimized by supplement use during winter in control group, whereas in case group due to lack of clear guidelines for this group and consequently low supplement use during long winters. Meanwhile, similar year-around levels of supplement intake between the case and control group (1000 IU), but different mean and median 25(OH)D may indicate different requirements for this specific clinical population who are suffering from SARD. Sainaghi *et al.* [19] evaluating 25(OH)D plasma concentration of 245 patients SARD and non-SARD groups, report the 750–1000 IU/day vitamin D might not be enough to provide minimum optimal 25(OH)D concentration of ≥75 nmol in this clinical population. Based on recent evidence, Holick [6] suggests daily vitamin D intake of 1500–2000 IU for healthy adults in general population. Most Canadian adults are not meeting this recommendation [20]. A recent study examined the effect of high-dose loading treatment in RA cases [21]. They reported a single oral dose of 300,000 IU followed by oral vitamin D3 of 800–1000 IU is effective in correcting hypovitaminosis D and PTH level [21]. The potential risks of high loading dose of vitamin D require further evaluations. In a randomized controlled trial, Sanders *et al.* [22] reported increased risk of falling in older women with annual dose of 500,000 IU vitamin D. Whether suppressing disease activity in SARDs requires more vitamin D, and the risks and benefits of high dose vitamin D requires more investigation in this clinical population.

Although many studies suggest the “overall” immuno-suppressive effect of vitamin D is SARDs, particularly RA, the Dietary Reference Intake Panel at the Institute of Medicine, responsible for setting recommendations for dietary intake, reported no conclusive evidence on the impact of vitamin D on SARDs [5]. The panel indicated that well-designed randomized controlled trials and large scale prospective cohort studies are needed to support the inverse relationship between vitamin D and autoimmune rheumatic diseases [5]. Since their report, more research was done. A meta-analyses reported vitamin D receptor polymorphism in RA and SLE, although only a few existing studies were analysed [21]. Others emphasised the impact of vitamin D on disease activity and elucidated the mechanisms [7,8,9,19,22]. Our evaluation of current evidence identified the following gaps in research:

1. Vitamin D receptor polymorphism and SARDs, and role of ethnicity in association between vitamin D and SARDs;
2. Differences in vitamin D requirements between SARD cases and the general population as well as the requirements for individuals who are at risk of SARD and individuals with established SARD;
3. Mechanisms of the impact of vitamin D in different pathways in immune system;
4. The potential interaction between vitamin D and drugs in SARD cases and optimal supplementation approaches.

Our study adds a Canadian perspective to existing literature on the impact of vitamin D on SARDs, mainly RD, however some limitations should be noted. The retrospective nature of our case-control study limited us to access information on other factors that might have impact on disease status such as smoking, physical activity, dietary intake, and sunlight exposure. Further, data on PTH plasma concentration and glucocorticoid use are not available. Sainaghi *et al.* reported high PTH level in RA patients irrespective of plasma vitamin D level [19]. Some medications such as glucocorticoids increase the destruction of vitamin D [23]. Finally, a single center study might under/over-estimate the hypovitaminosis D in outpatient rheumatology clinics.

## 5. Conclusions

Although more research is needed, the considerably low plasma 25(OH)D concentration in a considerably high number of SARDs outpatients (56%), as well as over five times higher risk of disease activity in RA cases with low plasma 25(OH)D concentration indicates the need for proper evaluation of vitamin D status in this clinical population. Further, appropriate training should be given to the patients to ensure the intake of the recommended amount of vitamin D per day through diet or supplement.
